# Elements in the Development of a Production Process for Modified Vaccinia Virus Ankara

**DOI:** 10.3390/microorganisms1010100

**Published:** 2013-11-01

**Authors:** Ingo Jordan, Verena Lohr, Yvonne Genzel, Udo Reichl, Volker Sandig

**Affiliations:** 1 ProBioGen AG, Goethestr. 54, 13086 Berlin, German; E-Mails: verena.lohr@probiogen.de (V.L.); volker.sandig@probiogen.de (V.S.); 2 Bioprocess Engineering, Max Planck Institute for Dynamics of Complex Technical Systems, Sandtorstr. 1, 39106 Magdeburg, Germany; E-Mails: genzel@mpi-magdeburg.mpg.de (Y.G.); ureichl@mpi-magdeburg.mpg.de (U.R.)

**Keywords:** AGE1.CR.pI, muscovy duck continuous cell line, modified vaccinia virus Ankara, MVA (modified vaccinia virus Ankara)

## Abstract

The production of several viral vaccines depends on chicken embryo fibroblasts or embryonated chicken eggs. To replace this logistically demanding substrate, we created continuous anatine suspension cell lines (CR and CR.pIX), developed chemically-defined media, and established production processes for different vaccine viruses. One of the processes investigated in greater detail was developed for modified vaccinia virus Ankara (MVA). MVA is highly attenuated for human recipients and an efficient vector for reactogenic expression of foreign genes. Because direct cell-to-cell spread is one important mechanism for vaccinia virus replication, cultivation of MVA in bioreactors is facilitated if cell aggregates are induced after infection. This dependency may be the mechanism behind our observation that a novel viral genotype (MVA-CR) accumulates with serial passage in suspension cultures. Sequencing of a major part of the genomic DNA of the new strain revealed point mutations in three genes. We hypothesize that these changes confer an advantage because they may allow a greater fraction of MVA-CR viruses to escape the host cells for infection of distant targets. Production and purification of MVA-based vaccines may be simplified by this combination of designed avian cell line, chemically defined media and the novel virus strain.

## 1. Introduction

Although the wide spectrum of current vaccines provides one of the most successful medicinal interventions available to health systems [1,2], infectious diseases still constitute an enormous global burden [3]. In particular, AIDS, tuberculosis, malaria and hepatitis C cause great suffering. Conventional killed vaccines against pathogens of these chronic infectious diseases have not been found to induce sterilizing immunity. It is hoped that methods additionally inducing cellular immunity may help in the fight against these indications [4,5,6]. Currently, the most efficient approach to induce cellular immunity is via use of attenuated pathogens. However, vaccination with live pathogens is associated with risks. They sometimes have the capacity to revert to virulent forms, or attenuation may be insufficient for immune-compromised recipients (e.g., [7,8]). Furthermore, especially for complex pathogens (such as the malaria protist) or extremely virulent pathogens (such as rabies virus), attenuated strains could not be generated or are considered to be too risky for use in humans. Vectored vaccines are being investigated to bridge the gap between the aim to induce a comprehensive immune response by simulating a genuine infection and the lack of suitable nonpathogenic forms of a disease agent. One very promising and versatile vector is modified vaccinia virus Ankara (MVA), a highly attenuated poxvirus for potential human recipients.

With MVA as an example in the center of a modern vaccine production process, this review summarizes the development from generation of a continuous cell line that is permissive for different vaccine viruses to the design of an efficient and scalable production process in chemically defined media. First results are also presented on what appears to be a genetic adaption of MVA to the highly controlled biopharmaceutical process conditions.

## 2. Modified Vaccinia Ankara

### 2.1. Viral Vectored Vaccines

Vectored vaccines are based on well-characterized infectious agents amenable to genetic manipulation and known to be stably attenuated [2,9,10]. Preferably, they are unable to replicate in the recipient so that they can be given even to immune-compromised patients in therapeutic, and not only prophylactic treatment regimes. Depending on the number and location of recipients in a vaccine program, production, supply, formulation and distribution of the vectors should be affordable, scalable and adaptable to the sometimes specific regional requirements or limitations. Furthermore, as vectored vaccines may have to be applied more than once, or are derived from agents that are naturally prevalent in the population, it is desirable that pre-existing immunity against the vector does not interfere with efficacy of the vectored vaccine. Availability of a vector that remains functional in presence of a reactivated immune response may also increase flexibility in heterologous prime-boost applications [9,11] where the same antigen is given consecutively, usually several weeks apart, via different methods or formulations.

Even closely related viruses (both as vectors and as pathogens) can differ substantially in their interaction with the immune system and the immune responses they elicit [9,12]. It can therefore be expected that a single vector will not combine all of the properties required for the diverse vaccine applications. Gene therapy vectors that are designed to maintain prolonged expression of transgenes with minimum damage to the host cell may not be suitable as vaccine vectors if they induce immune tolerance [13,14]. However, vectors optimized for gene therapy can be used in vaccination regimes, for example to enhance the efficacy of whole tumor-cell vaccines via expression of immune modulators [15]. The wide spectrum of recombinant viruses [14,16] investigated for immunization against infectious and neoplastic diseases includes sendai virus, alphaviruses (especially those attenuated by chimerization of different species), measles virus, adenoviruses of different animal species, nonintegrative lentiviral vectors, vesicular stomatitis virus, and poxviruses (vaccinia virus, MVA, and avian poxviruses).

### 2.2. Properties and Challenges Associated with MVA (Modified Vaccinia Virus Ankara)

MVA is derived from a vaccinia virus, a member of the *Poxviridae*, subfamily *Chordopoxvirinae* (the other subfamily, *Entomopoxvirinae*, contains viruses that infect insects). Poxvirus particles are enveloped and resemble flattened barrels approximately 260 nm in length [17]. They enclose a single segment of double stranded DNA that ranges in size between 139 kbp and 307 kbp and contains 178–334 open reading frames, 90 therefrom shared among poxviruses infecting vertebrates [18,19,20,21,22]. The large number of genes also includes the coding sequences for enzymatic functions that allow replication in the cytoplasm, which is an unusual property among the DNA viruses.

Poxviruses that are highly attenuated for humans are very promising vaccine vectors that naturally infect only birds (for example, canarypox strain ALVAC and fowlpox strains FP9 and TROVAC, both of the *Avipox* genus of the *Poxviridae*) or have been adapted experimentally to avian cells (MVA, *Orthopoxvirus* genus). They are investigated in primer-boost regimes against infectious and neoplastic diseases that range from AIDS [23,24], malaria [25,26], and tuberculosis [27] to prostate cancer [28] and melanoma [29].

The immediate ancestor of MVA is the strain chorioallantois vaccinia virus Ankara (CVA) [30]. CVA is derived from vaccinia virus and was maintained as a smallpox vaccine by alternating passages in the skin of calf and donkeys at the Turkish vaccine institute in Ankara. A sample of this CVA strain was transferred to Germany in 1953 where it was attenuated by serial passaging on chicken-derived material as production substrate [31]. The plaque-purified isolate of passage 516 was called MVA and was subsequently shown to have lost approximately 15% of its genome at multiple sites in the course of this attenuation [21,32]. The six deletion sites together with additional minor disruptions in several genes of MVA contribute to a severe constriction of the host range with the result that, as opposed to parental CVA, replication of MVA in human cells is blocked or severely impaired [33,34,35]. However, although MVA cannot replicate even in immunocompromized human patients, the viral genes (and genetic payload of recombinant viruses) is expressed very efficiently and results in a robust T-cell mediated immune response [36,37,38,39] that is not inhibited by preexisting immunity [40]. Vaccinia viruses can be manipulated genetically by homologous recombination and accept inserts of at least 25,000 bp [36,41,42].

Safety of MVA for use in humans has been demonstrated in the final years of the smallpox eradication campaign of the World Health Organization and, more recently, in numerous clinical trials (e.g., [5,24,26,43,44]). However, while host restriction increases safety it is also associated with challenges. Vectors that are highly attenuated amplify only to very low levels or not at all at the site of injection and therefore have to be given at high doses for optimal stimulation of the immune system [45]. Production of the required volumes of concentrated poxviruses depends on avian host cells. Since the 1930s and until today, vaccines adapted to avian substrates are being produced in primary cells obtained from embryonated chicken eggs [46,47]. However, dependence on animal-derived material with a limited life span that has to be continuously fed into vaccine production processes is not an optimal situation. In addition, primary cells are difficult to adapt to advanced cultivation strategies in modern bioreactors.

## 3. A New Cell Substrate

### 3.1. Avian Finite and Continuous Cell Substrates

Contrary to the common human diploid finite cell lines, WI-38 and MRC-5 [48], no cell banks appear to be available of the corresponding avian cell substrates. Embryonated chicken eggs are an obviously complex material. However, also primary chicken embryo fibroblasts have to be introduced continuously into the production process. This property is one considerable disadvantage of an otherwise versatile and well-characterized substrate that is currently in use for production of vaccines against rabies, measles, mumps, yellow fever and influenza. Eggs of a well-defined regulatory status referred to as *Specific Pathogen Free* (SPF) are preferred for most vaccine production processes. If embryonated eggs of a *vaccine-quality non-SPF* status are used, additional adventitious agents testing may have to be performed during the vaccine development and production process. Maintenance of SPF status requires strict isolation of the flocks from environmental contaminants, personnel that showers and changes into protective clothing, and treatment of food and air prior to contact with the animals. Furthermore, animals are screened for at least eighteen pathogens and no medication that may interfere with the sensitivity of detection should be administered. Supply constraints have been reported [49,50] because there are not too many facilities that can provide pathogen-free eggs.

Another disadvantage of production with primary cells is that an increased effort is necessary to perform scalable suspension processes. Because of the complex logistics associated with embryonated eggs, transportable processes for implementation at multiple sites and in newly industrialized countries may also be difficult to realize with such a substrate.

### 3.2. Duck *versus* Chicken

To advance production of vaccine strains adapted to replication in avian cells, we have generated a continuous cell line, AGE1.CR.pIX (CR.pIX thereafter in this text), derived from primary muscovy duck (*Cairina moschata*) explants [51]. Our decision to provide a duck (rather than chicken) cell line was associated with scientific uncertainties regarding the host range of the viruses that may have to be produced on the corresponding cell line (especially MVA and other host-restricted poxviruses), and with regulatory risks regarding acceptance of such a cell line. However, although available literature mostly discusses chicken host cells, we avoided galline primary material. This decision was motivated by reports on particles derived from endogenous retroviruses that may contaminate vaccines produced with the help of embryonated chicken eggs [52,53]. Endogenous retroviruses are proviruses that have integrated into the germ line of their host and are common in vertebrates, including chickens and humans, where they account for up to 3% and up to 8% of the host genomic sequences, respectively [54,55,56,57]. Recently, well after initiation or our program in 2004, the full genome of a duck of the *Anas* genus has been sequenced [58] and a proviral genome content of approximately 1% has been determined.

**Figure 1:**
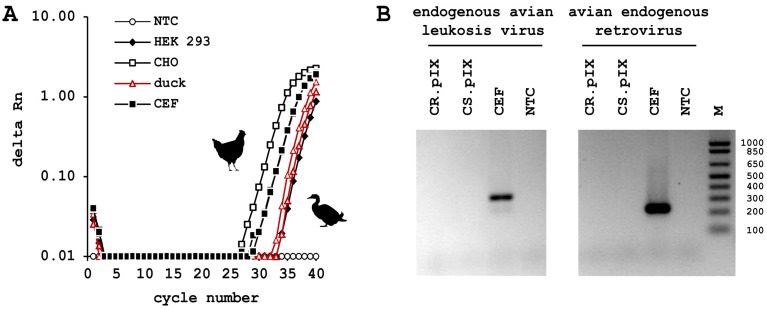
(**A**) Test on particle-associated reverse transcriptase activity in culture supernatant. Product Enhanced Reverse Transcriptase PCR (PERT-PCR) was performed with Brome mosaic virus RNA activated with primer 5′-GCCTTTGAGAGTTACTCTTTG-3′ as tester, and primers 5′-AAACACTGTACGGCACCCGCATT-3′ and 5′-GCCTTTGAGAGTTACTCTTTG-3′ to amplify any cDNA that may have been generated by reverse transcriptases in the sample; (**B**) PCR against the chicken endogenous proviruses was performed with primers 5′-TGGTGACCCCGACGTGAT-3′ and either 5′-CCTATTTCCTTCTTAGAAGGAGA-3′ (EAV) or 5′-CGATCTCCTTCCCGGAAGGAGT-3′ (ALV) as reverse primers. CS.pIX: cell line obtained from muscovy duck somites; NTC: non-template control.

However, the majority of these inheritable and sometimes mobile elements is defective at multiple locations and cannot form functional particles. Assays on the presence of reverse transcriptase help to differentiate potentially infective endogenous and exogenous retroviral particles from defective particles (Figure 1). To detect this enzymatic activity the particles are first collected by ultracentrifugation of a cell-free sample. This step increases sensitivity but is also important to remove legitimate reverse transcriptase activity that, for example, can be found in cellular DNA repair enzymes. Lysis buffer is added and if particles are present reverse transcriptase is released. Next, this solution is incubated together with nucleotides and a defined RNA molecule annealed to a primer in appropriate reaction buffers. If particle-associated reverse transcriptase is present then a known cDNA is generated that subsequently can be detected by PCR. Application of this Product Enhanced Reverse Transcriptase PCR (PERT-PCR) has revealed contamination of released vaccine lots that were produced on chicken material [52,53]. However, the retroviruses derived from chicken have not been associated with any risk to human recipients. As measurable benefit of vaccines outweigh theoretical risk from the endogenous retroviruses the WHO recommended in 1998 [59] that production of vaccines should continue in chicken-derived material.

We hoped to be able to completely avoid the problem of potential contamination with endogenous retroviral particles by immortalization of avian cells from a species that may not be susceptible to some of the exogenous retroviruses that infect chickens [60]. The literature also suggested that endogenous retroviruses that contaminate vaccines today may have entered the chicken germ line after the *Galliformes* and *Anseriformes* have separated on the phylogenetic tree [61]. A direct ancestor along the development path of the CR.pIX continuous cell line was tested by PERT-PCR and shown no to release particle-associated reverse transcriptase above the limit of detection (Figure 1). The results have been confirmed independently by others with CR.pIX Research and Master Cell Banks.

In addition to assays on retroviral activity, the CR.pIX cell banks have also been tested against avian, bovine and porcine pathogens by transmission electron microscopy for detection of microbial structures, real time PCR against specific pathogens including circoviruses, cocultivation with different indicator cell lines for cytopathic effects caused by unknown pathogens, inoculation of cell line material into various agars and broths to test against mycoplasma and other bacteria, inoculation of chicks for antibody induction, and inoculaton of embryonated eggs, guinea pigs and adult and suckling mice for any signs of infection. It is yet another advantage of continuous cell lines compared to primary chicken cells that such tests can be performed ahead of production on cell banks that serve as a characterized source for multiple substrate seeds. Assays against contamination of vaccines produced on primary cells have to be performed on the final bulk because the time between collection of embryonated eggs and production of vaccines is short. Occasionally, complete vaccine lots have to be discarded if the SPF status was compromised [49]. Furthermore, these complex assays have to be performed repeatedly, which is expensive and subject to risk of sudden regulatory changes [62]. All steps for the CR.pIX development were performed in a clean-room environment. Each manipulation and culture passage, as well as all solutions or buffers that came into contact with the cell line, are fully documented.

### 3.3. CR.pIX

One important reason for a slow adoption of continuous cell lines for vaccine production may be that health regulatory properties and consequences are not yet fully defined for such substrates. The main concern associated with continuous cell lines is that host cell-derived oncogenic components may be transferred to vaccine recipients. Because especially DNA coding for transforming factors is considered to constitute a risk, the amount of DNA allowed in an injectable vaccine is currently limited to 10 ng per dose [63]. This value has been derived by a defined-risks calculation that relates the average DNA fragment size contained in a vaccine preparation to the size of the average oncogene coding sequence, the size of the host cell’s genome, and the expected number of oncogenes within the genome required to be transferred for an effect [64]. Most likely this number overestimates the risk as no tumors were observed in a longitudinal study with monkeys that were injected with up to 1000 μg of DNA derived from a continuous human cell line [65].

Confidence in the defined-risks calculation is proportional to the level of knowledge about derivation and properties of a continuous cell line. To facilitate acceptance of the CR.pIX cell line, we chose to immortalize the primary cells with the E1 region of human adenovirus 5. As described previously [51], retina cells were isolated from a single duck embryo. The *E1A* and *E1B* genes were introduced into the cells by liposomal transfection of an expression plasmid without exogenous (antibiotic) selection. Immunofluorescence assays of cultures at various passage levels demonstrated that only cells that stably expressed the *E1A* and *E1B* genes survived beyond senescence, confirming the intended induced (as opposed to a spontaneous) immortalization. The final step in the cell line development program, adaptation to proliferation without anchorage, was performed by cultivation in commercial media designed for human suspension cell lines [66].

Several advantages are associated with immortalization using the adenovirus E1 region.

(1) There is precedence for this method in the HEK 293 [67] and PER.C6 human cell lines [65,68] that have already been discussed by authorities (for example, document 3750b1 in [69]).
(2) Human adenoviruses are both endemic and pandemic pathogens responsible for 2%–7% of upper respiratory infections in children. Although they have also the capacity to cause severe disease especially in immune-compromised patients [70] they are not associated with human tumors [71].
(3) Immortalization is the result of a concerted action of distinct and separate E1A and E1B gene products. This synergy necessitates concurrent events for experimental immortalization but also increases the probability against accidental tumor induction by a contamination.
(4) That many of the biochemical pathways leading to immortalization by the E1 region are known may further facilitate defined-risks estimates. Briefly, by relieving inhibition of the E2F transcription factors, the E1A protein promotes cell cycle progression. However, various parallel mechanisms, including induction of DNA damage responses and augmented expression of an alternate reading frame (ARF) of the *INK4a/ARF* gene locus, lead into activation of apoptosis [71]. One pro-apoptotic signaling cascade is mediated by the gatekeeper of the genome, the p53 protein. This central node must be controlled by the viral *E1B 55K* gene product for efficient adenovirus replication [72]. Another chain of events occurs at the mitochondrial membrane. Inhibition of this pathway, that is modulated by caspase proteolysis, is exerted by the homolog to cellular Bcl2 proteins, the viral *E1B 19K* gene product [72,73]. The surprising property of E1A protein to sensitize cells for apoptotic stimuli and to suppress proliferation of some tumors [71,74] is investigated as therapeutic option in virotherapy [75] and *E1A* gene therapy for cancer patients [74,76,77].
(5) For certain applications there may also be a virological advantage if E1 proteins are expressed in a host cell. Some vaccine strains are attenuated via lesions in factors that allow the virus to mask itself against the innate immunity of the cell. E1A has also been implicated in the viral defense against antiviral responses [78,79]. Although antiviral defenses within cells are activated and mediated by multiple, partially overlapping pathways [80], replication of some attenuated viruses may benefit and yields of infectious units may increase if the cellular defense is already repressed at some nodes of the redundant pathways.

The CR cell line was further modified by transfection of an expression plasmid for the adenovirus pIX structural protein to obtain the CR.pIX cell line. We hypothesize that some viruses, even if they are not related to adenoviruses, benefit from the presence of pIX because this protein may constitutively activate Hsp90, a central factor in heat shock responses [51]. Replication of a virus can impose a significant metabolic burden on the host cell that may also impact proper processing and folding of the structural proteins and viral replication machinery. Activation of chaperones in the heat shock cascade may alleviate such constrains and increase the yield of infectious particles for some viruses.

## 4. Application

### 4.1. Process Development

**Figure 2:**
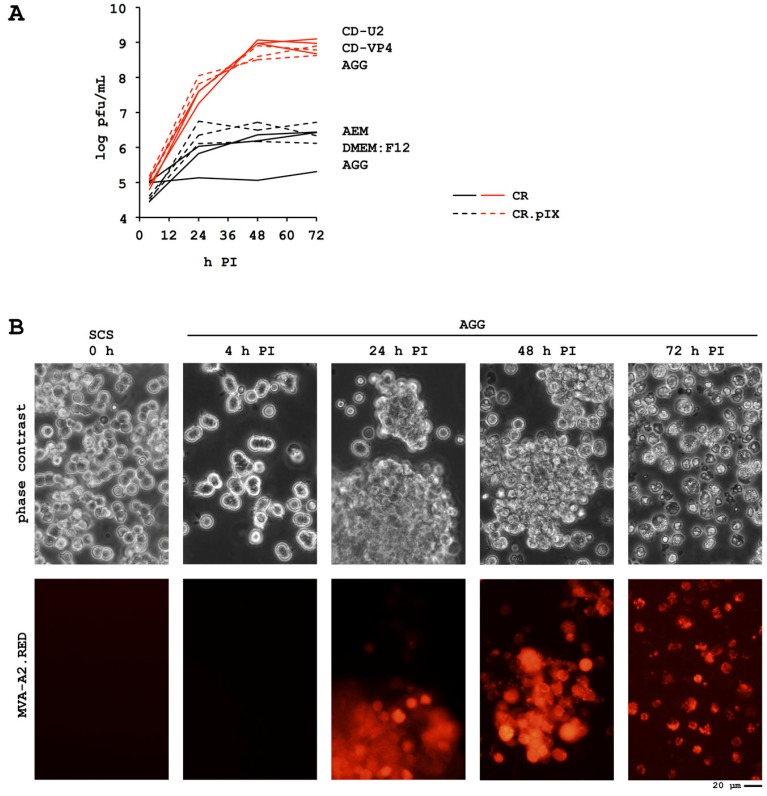
(**A**) Chemically defined media and induction of cell aggregates (AGG) for increased yields of MVA (modified vaccinia virus Ankara). Adenovirus Expression Medium (AEM, Invitrogen, Carlsbad, CA, USA) allowed excellent cell proliferation but even together with DMEM:F12-induced AGG the MVA replication was insufficient. Infectious titers in chemically-defined CD-U2 and CD-VP4 media (both produced by Biochrom, Berlin, Germany) are consistently beyond 10^8^ pfu/mL after infection of 2 × 10^6^ cells/mL with a MOI of 0.05 to 0.1; (**B**) Single-cell cultures (SCS) are maintained in CD-U2 or CD-U3 (Biochrom). AGG were induced by addition of CD-VP4 and a recombinant MVA that expresses DsRed1 from within deletion site III was added to a MOI of 0.05. Virus spreads within infected aggregates that start to disintegrate 48 h PI due to CPE. PI, post infection.

The adherent CR and CR.pIX cell lines were adapted to proliferation in suspension and tested for propagation of a wide spectrum of human and veterinary vaccine viruses [66,81,82]. Surprisingly, although replication of MVA in adherent CR.pIX monolayers is efficient with yields above 10^8^ pfu/mL within 48–72 h post infection, peak titers were variable and often below 10^7^ pfu/mL in suspension cultures [51,66]. Results from a series of infection experiments with adherent cultures in presence of suspension media and suspension cultures in different basal media indicated that one reason for the difficulties might be the large fraction of infectious virions remaining cell-associated during poxvirus replication [83]. For this reason, we developed complementary chemically-defined media formulations: a cell proliferation medium for single-cell cultivation in routine maintenance and expansion of CR.pIX cells in stirred-tank as well as in wave bioreactors, and a virus production medium that is added at the time of infection. The virus production medium induces cell aggregates that we hypothesize should allow efficient replication of viruses with a tendency to spread by direct cell-to-cell contact (Figure 2). This process is robust and consistently gave yields beyond 10^8^ pfu of MVA/mL at cell densities between 0.5 × 10^6^ and 2.5 × 10^6^ cells/mL and MOI between 0.01 and 0.1 [66]. This procedure is also fully scalable and was demonstrated in bioreactors up to 200 L working volume for a recombinant MVA vaccine candidate [84].

### 4.2. Metabolic Properties

Having developed a new cell line for production of vaccines in bioreactors, investigation of metabolic properties was an early research topic. One motivation for these studies was that although cells of avian origin are being used for derivation and production of vaccine strains since the 1930s [46,85], some metabolic characteristics have not been studied in detail. Mammals and birds can differ in some aspects both at the level of the organism and at the level of explanted cells [58,86,87,88], and it cannot be excluded that an unexpected metabolic property may be important for design and optimization of biotechnological applications. 

Conversely, it is possible that CR.pIX cell lines show the metabolic characteristics expected of continuous vertebrate cell lines such as HEK 293 or CHO, irrespective of their non-mammalian origin. Such a property would facilitate medium and process optimization and may contribute to the study of evolutionary conserved biochemical pathways.

**Figure 3:**
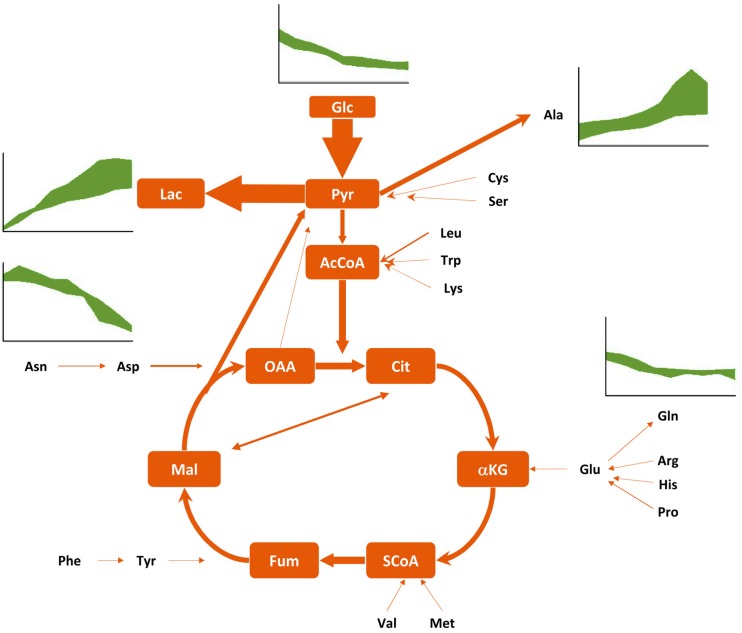
Schematic flux distribution of the central metabolism of CR.pIX cells, including glycolysis, citric acid cycle and amino acid catabolism. Selected concentration profiles of extracellular metabolites indicate uptake and release of main metabolites (data bands include several experiments). Intracellular fluxes are depicted with arrow with sizes corresponding to the rate of the illustrated reaction. Glucose (Glc), lactate (Lac) and glutamine (Gln) were determined with a Bioprofile 100 Plus (Nova Biomedical, Waltham, MA, USA), extracellular amino acid concentrations were determined by HPLC. Abbreviations: Amino acids as three letter code; Pyr, pyruvate; AcCoA, acetyl coenzyme A; Cit, citric acid; α-KG, α-ketoglutarate; SCoA, succinyl CoA; Fum, fumarate; Mal, malate; OAA, oxaloacetate.

Indeed, extensive analyses of CR.pIX cell cultures in different cultivation systems combined with metabolic flux analyses demonstrated that some metabolic properties of this duck-derived cell line are similar to previously characterized mammalian and even insect cell lines. For example, CR.pIX cells share an overflow metabolism fueled by glucose consumption and terminating predominantely in lactate production and release (Figure 3). The energy-producing citric acid cycle is fed not only directly via glycolysis, but is also supported by amino acid catabolism towards TCA cycle intermediates with one side effect that ammonia concentrations never reached critical concentrations of more than 2 mM.

Extracellular lactate concentrations remained typically below growth-limiting levels of 40 mM. However, for cultivations that either target high cell concentrations or long cultivation intervals pH control is required to compensate lactate release. Current metabolic and media optimization studies focus on lowering lactate consumption, on improving efficiency of the citric acid cycle and on reaching high cell densities (>2 × 10^7^ cells/mL). The aim of this development is to stabilize long term cultivation of infected cultures in bioreactors, which in turn may increase yields for some vaccines and can extend harvest intervals [89,90]. Implementation of such a process may be feasible with MVA because one obstacle in long-term cultivation, the potential emergence of defective interfering particles (DIPs) [91,92,93], appears not to affect poxviruses. However, the requirement for aggregate induction specific to MVA replication complicates long-term cultivation of the host cells. It is also in this context that the novel MVA genotype described in the next section may find a useful application.

### 4.3. A Novel MVA Genotype

When we determined the growth kinetics of MVA preparations obtained from serial passages in the suspension culture system, an increase in titers was observed with increasing passage number [94]. The motivation for this experiment was to study whether MVA populations or isolates with different phenotypes could be obtained: MVA was rendered host-restricted by repeated passaging in chicken cells (whereas we produce MVA in a duck cell line), and the biotechnologically advantageous single-cell suspension environment in chemically defined medium is different from replication in tissue (consisting of densely packed cells of different lineages perfused by serum). As we expected that an explanation for this phenomenon will be visible by changes within the genome, we isolated viral DNA from preparations of passages 2, 7 and 11 and determined the genetic sequence of the respective virus populations. Interestingly, only three point mutations were observed [94] localized in three genes encoding the structural proteins, MVA114L (A3L in the terminology describing vaccinia virus Copenhagen), MVA120L (A9L) and MVA145R (A34R). All three mutations are coding and replace His_639_ in protein A3 to Tyr, Lys_223_ in A9 to Glu, and Asp_86_ in A34 to Tyr. The genotype with the three mutations readily accumulated in subsequent passages in suspension culture and was further plaque-purified to obtain a pure isolate. The thus derived strain was called MVA-CR and the first pure isolate was called MVA-CR19.

Vaccinia virions consist of a compact nucleoprotein core and up to three membranes. The three affected genes are components of different layers of the complex viral particles. A3 and A9 are embedded in the interior of the virion. The A3 protein appears to contribute to correct condensation of the genomic DNA and formation of the core [95,96,97], and A9 is proposed to be part of the matrix that connects the core to the innermost envelope [98].

This first envelope is obtained from host-derived lipids at dedicated assembly sites in the cytoplasm called viral factories (reviewed in [99,100]). The mature infectious viruses (MVs) that are released from the viral factories can remain within the cell or be transported along microtubules towards the plasma membrane. A fraction of the MVs are wrapped in vesicles of the trans-Golgi network in such a way that a double membrane (separated by the lumen of the former vesicle) forms the new shell of the particles that now are equipped with three membranes. The most distal membrane can fuse with the plasma membrane so that a doubly-enveloped virion is attached on the outside of the host cell. Only a fraction of these cell-associated viruses are released as extracellular viruses (EVs). The outer membrane of the EVs disintegrates to release the MVs at the time of reinfection. The A34R protein, which is also mutated in MVA-CR, is described in the literature to contribute to the release of EVs and exposure of MVs [83,101,102,103,104].

**Figure 4:**
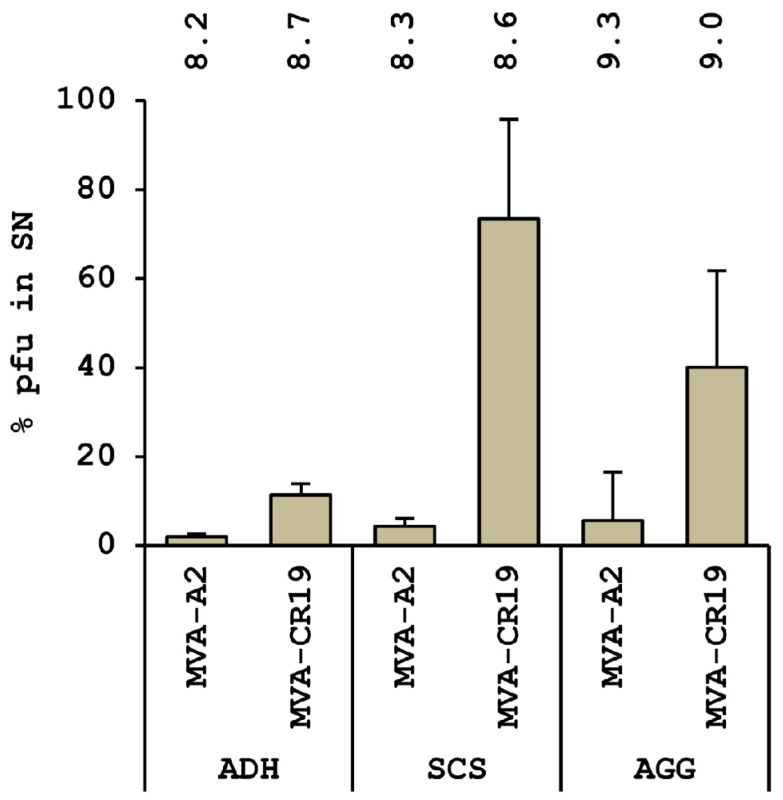
A higher percentage of infectious units is observed in the cell-free supernatant (SN) in cultures infected with MVA-CR19. MVA-A2: wildtype MVA; ADH: adherent CR.pIX culture maintained in DMEM:F12 medium containing 5% bovine serum; SCS: single-cell culture in CD-U3 medium; AGG: aggregate suspension culture in CD-U3 and CD-VP4 media. Number on top of the column gives log pfu/mL in the complete lysate. Error bars give standard deviation obtained in three experiments.

After recovery of a pure isolate of the new genotype, yields and replication kinetics of MVA-CR and wildtype MVA were compared in single-cell and aggregate suspension cultures in chemically defined media, and on adherent monolayers in basal medium supplemented with bovine serum [94]. A larger fraction of extracellular infectious units was detected in these experiments in suspension cultures infected with MVA-CR compared to the wildtype reference (Figure 4). This property may be important in the implementation of production options utilizing continuous processes [91].

**Figure 5:**
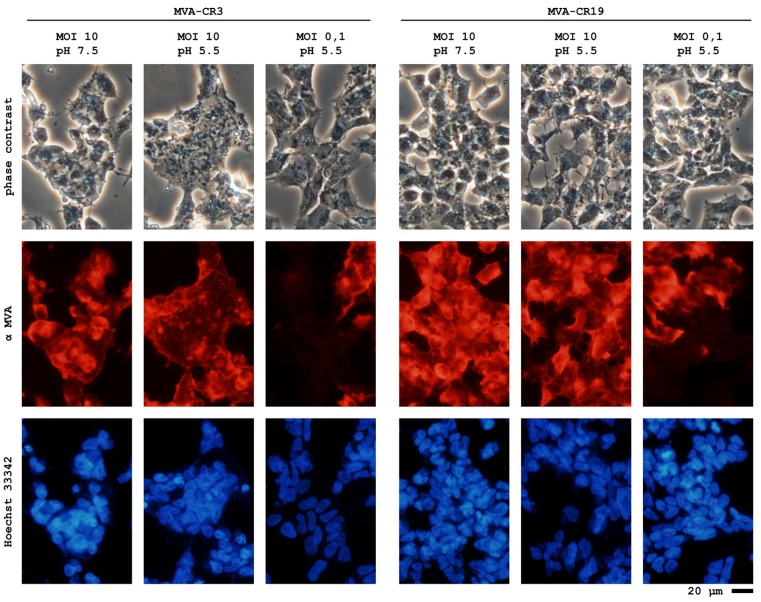
Syncitia formation appears to be more pronounced in adherent cell monolayers of CR.pIX cells infected with wildtype MVA compared to MVA-CR19. CR.pIX adherent cell monolayers were infected with MVA with a MOI of 10 and 0.1 as a control. The fusion apparatus of MVA was further activated by a brief pH shock [105]. Cells were fixed after 22 h with 2% formaldehyde and immunostained with antibodies against MVA. MVA-CR3, early passage preparation consisting of wildtype MVA without a detectable MVA-CR genotype; MVA-CR19, plaque-purified MVA-CR viruses; α MVA, antibody immunostaining; Hoechst 33342, live stain of DNA in cell nuclei.

We hypothesized that mutations in MVA-CR may facilitate release of viruses with this genotype. Alternative explanations for this effect may be an improved infectivity of MVA-CR or faster maturation of viral particles. To study whether virions detach more easily from the host cell, we compared induction of syncitia in monolayers infected with MVA-CR19 or an earlier passage of an MVA population, MVA-CR3, where the majority of the viruses still is wildtype (Figure 5). Syncitia can be formed if the viral machinery responsible for cell entry induces the fusion of the plasma membranes of neighboring cells. We would therefore expect that, if a greater number of infectious particles remain cell associated, then the probability will be greater as the number of viral fusion components is above a threshold conducive for syncitia formation. As shown in Figure 5, syncitia are readily observed in monolayers infected with wildtype MVA, whereas fewer such events appear in monolayers infected with MVA-CR19. This result is consistent with the hypothesis that the MVA-CR phenotype of increased infectious activity in the extracellular volume could be due to an increased tendency of this virus to escape the host cell.

### 4.4. Current Status

One limitation in the previous experiments is that three mutations have been detected concurrently in MVA-CR19. We do not know whether all three mutations need to cooperate to yield the observed phenotype, and whether the phenotype does indeed reflect enhanced escape of the infectious units from the host cell. We can also not be certain that additional mutations and genomic alterations that may have been missed in the sequence evaluation contribute to the observed properties of the new MVA strain. To address some of these questions, we currently assemble the three mutations in different combinations into the wildtype genome. For improved detection and quantification of the recombinant viruses, we also inserted *EGFP* and *DsRed1* reporter genes under control of a hybrid early/late MVA promoter into deletion site III of the different MVA instances [41,106].

**Figure 6:**
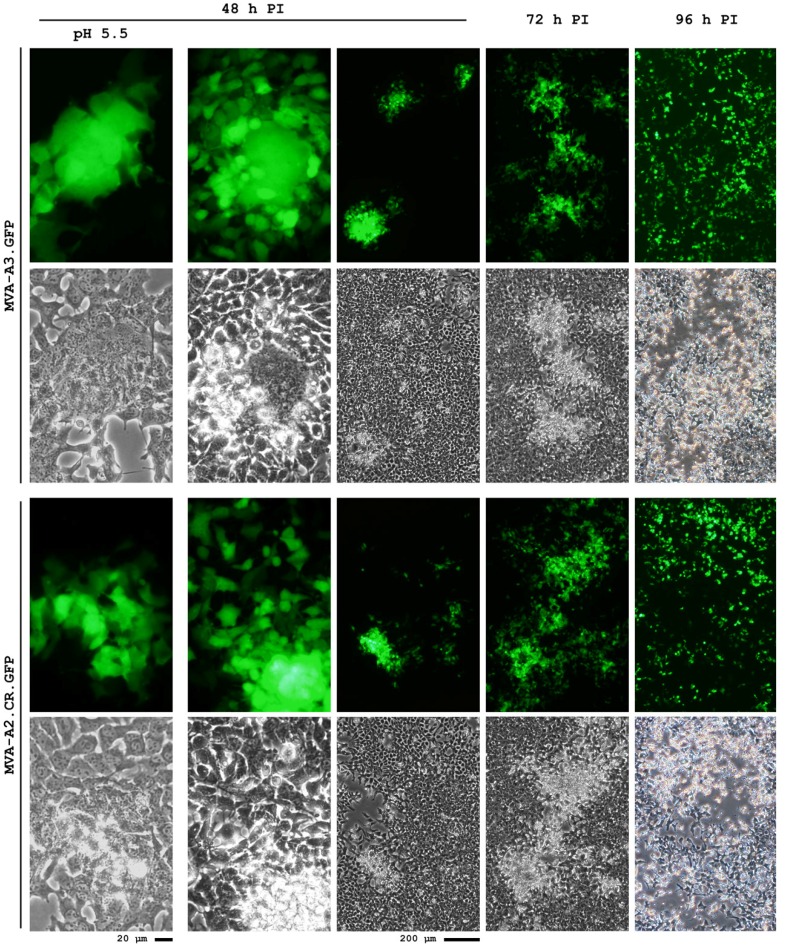
Plaques of recombinant virus containing the MVA-CR genotype mutations in the backbone of wildtype virus exhibit a larger fraction of scattered cells and fewer spontaneous syncitia. Synthetic DNA containing the mutations of the MVA-CR genotype in the three genes were recombined into a wildtype MVA, together with GFP as reporter into deletion site III, obtaining the virus MVA-A2.CR.GFP. A wildtype MVA that also contains GFP in deletion site III is designated as MVA-A3.GFP. CR.pIX cell monolayers were infected with a MOI of 0.01. Column marked with pH 5.5 header denotes syncitia induction by pH shock at 30 h post infection.

As shown in Figure 6, first experiments confirm that some combinations of reverse engineered viruses exhibited a less confined plaque phenotype and reduced spontaneous syncitia formation compared to the ancestral wildtype that was also equipped with a reporter gene in deletion site III. This result is consistent with our hypothesis that MVA mobility in infected cells may have been affected by the mutations that were selected during passaging in the suspension cultures.

Another open question is whether reactogenicity and attenuation of MVA-CR viruses may have been modulated by the mutations. An important mechanism underlying the attenuation phenotype of MVA in human cells appears to be a block during assembly of infectious particles [107,108]. Early morphogenesis precedes the later steps catalysed by A34, but A3 and A9 are involved in the transition of non-infectious to infectious virus. Mutations of these two proteins may therefore affect the progression of maturation in non-avian host cells. Although there are no reports suggesting that A3 and A9 contribute to virulence of vaccinia viruses, additional studies may be required to characterize MVA-CR. The expected host cell restriction, mainly, no measurable replication of MVA-CR19 in human HEK 293 and simian Vero cells, has been confirmed previously [94].

## 5. Conclusions

In this review, we have focused on one approach for production of a promising group of viral vectors, poxviruses attenuated by host-restriction. Novel avian cell lines permissive for these poxviruses have been generated with focus on conformity with regulatory guidelines. The CR and CR.pIX cell lines of this program were adapted to proliferation in suspension cultures, and chemically-defined media were developed to minimize risks to vaccine recipients and to improve lot-to-lot consistency. A considerable fraction of infectious virions remain attached to the infected cell. To improve virus replication in suspension cultures, the process was refined to facilitate cell-to-cell spread of virions via induction of cell aggregate formation. In this system, a novel MVA genotype (MVA-CR) was discovered that we believe has advantageous properties for suspension cell culture-derived vaccine manufacturing. Production and purification of MVA independent of primary chicken embryo fibroblasts is associated with both advantages and significant challenges [66,109,110]. One advantage of the new genotype may be to facilitate high cell-density vaccine production processes including continuous processes, so that greater volumes of affordable injectable vaccine preparations can be obtained. MVA-CR is currently studied in greater detail to explain the ability for replication in single-cell suspensions and to critically assess advantages or disadvantages for clinical application.
